# CREM Alpha Enhances IL-21 Production in T Cells *In Vivo* and *In Vitro*

**DOI:** 10.3389/fimmu.2016.00618

**Published:** 2016-12-19

**Authors:** Kim Ohl, Anastasia Wiener, Ralph Lippe, Angela Schippers, Carolin Zorn, Johannes Roth, Norbert Wagner, Klaus Tenbrock

**Affiliations:** ^1^Pediatric Immunology, Department of Pediatrics, RWTH Aachen University, Aachen, Germany; ^2^Institute of Immunology, University of Münster, Münster, Germany; ^3^Institute of Biochemistry and Molecular Immunology, RWTH Aachen University, Aachen, Germany

**Keywords:** SLE, autoimmunity, CREM, CREB, NFAT, IL-21

## Abstract

The cAMP-responsive element modulator alpha (CREMα) plays a role in autoimmunity and, in particular, in systemic lupus erythematosus. CREMα negatively regulates IL-2 transcription and activates IL-17 expression by direct transcriptional mechanisms. To understand the role of CREM in autoimmunity, we recently generated a mouse with a transgenic overexpression of CREMα selectively in T cells. This mouse is characterized by enhanced IL-17 and IL-21 expression. We, herein, dissect the transcriptional mechanisms of enhanced IL-21 transcription in these mice. T cells of CREMα transgenic mice display an enhanced binding of CREMα to the CD3ζ chain promoter resulting in decreased CD3ζ chain expression. This is accompanied by a decreased excitation threshold and enhanced Ca^2+^ influx, which is known to induce IL-21 expression *via* NFATc2 activation. However, CREMα directly binds to cAMP-response element (CRE) half-site within the *Il-21* promoter, which results in enhanced promoter activity shown by promoter reporter assays. CREMα-induced IL-21 transcription is not abrogated in the presence of cyclosporine A but depends on an intact CRE site within the IL-21 promoter, which suggests that CREM largely enhances IL-21 expression by direct transcriptional regulation. IL-21 transcription is critical for IL-17 generation in these mice, since IL-21 receptor blockade downregulates IL-17 transcription to wild-type levels. Finally, this is of functional relevance since CREMα transgenic mice display enhanced disease activity in dextran sodium sulfate-induced colitis accompanied by higher local IL-21 expression. Thus, we describe two novel mechanisms of CREMα-dependent IL-21 transcription. Since T cells of systemic lupus erythematosus patients are characterized by enhanced IL-21 transcription, this might also be of functional relevance in humans.

## Introduction

cAMP-responsive element modulator (CREM) is a member of the ATF/CREB type bZip transcription factors family. cAMP activates proteinkinase A that phosphorylates and thus activates CREB and CREM. Members of the ATF/CREB family bind to the cAMP-response element (CRE) in the promoter regions of target genes. This binding results in either suppression or activation of promoter activity, and, respectively, of gene expression (1, 2). CREMα, a CREM isoform generated by alternative splicing, has key functions as an epigenetic and transcriptional regulator of cytokine expression in T cells from systemic lupus erythematosus (SLE) patients (3). T cells from patients with SLE exhibit CREMα overexpression (4). CREMα contributes to silencing of *IL2* in these cells through transrepression and tissue- and region-specific recruitment of specific DNA and histone methyltransferases or HDACs (5). In addition, CREMα transactivates *IL17a* promoters, which suggests that CREMα contributes to increased *IL17a* mRNA expression and IL17a protein levels in SLE patients (6). Notably, the observed effects of CREMα on IL-2 and IL-17a cytokine production in humans are also observed in transgenic mice with T cell-specific CREMα overexpression (under control of the *cd2* promoter) [CREMα transgenic (tg)] (7). These mice have decreased IL-2 and increased IL-17a levels and are more prone to develop signs of autoimmunity (including lymphadenopathy and higher autoantibody titers against double-stranded DNA) when an additional genetic deletion of the *cd95* gene (Fas) is present (7, 8).

IL-21 is a type I cytokine, which exerts critical roles in immune cell differentiation and function by signaling through a heterodimeric receptor, which is formed by common gamma chain (shared with IL-2, IL-4, IL-7, IL-9, IL-13, and IL-15 receptors) and an IL-21-specific receptor (IL-21R) (9, 10). Since IL-21R is expressed on CD4^+^, CD8^+^ T cells, B cells, NK cells, dendritic cells, macrophages, and also non-immune cells (e.g., fibroblasts, epithelial cells, and keratinocytes) (10, 11), IL-21 regulates multiple cell types during the course of inflammatory responses. IL-21 is produced by activated (NK) T cells and by differentiated CD4^+^ T cell subsets (12). Of those, Th17 cells are the main producer of IL-21 in mice (13, 14) and IL-21 plays a key role in the amplification of Th17 cell responses (13). Furthermore, IL-21 is produced by and plays a role in development of follicular T helper cells by inducing Bcl-6 expression (15, 16). In B cells, IL-21 promotes plasma cell differentiation (17–19). With regard to other T cell subsets, IL-21 suppresses the differentiation and functions of Th2 cells (20) and negatively regulates induced regulatory T cells as IL-21 antagonizes TGF-β1-mediated induction of FoxP3-expressing T cell (13, 21). Moreover, IL-21 renders human CD4^+^CD25^−^ T cells resistant to the suppressive effects of regulatory T cells (22).

Expression of IL-21 is strictly calcium dependent, which is mediated by NFATc2 binding to the IL-21 promoter region (23). NFAT activation is induced by calcium signaling as NFAT proteins are activated *via* phosphorylation by the by calcium/calmodulin-dependent phosphate calcineurin, thereby translocating NFAT proteins from the cytoplasm to the nucleus (24). The central role of NFAT for TCR-stimulated *Il-21* promoter activity was further confirmed by Mehta et al. (25). However, IL-21 is still expressed in NFATc2-deficient mice, implying that additional transcription factors are be involved in the regulatory events of *il21* gene expression (23). One of these transcription factors is c-Rel. This member of the NFκB transcription factor family activates IL-21 expression *via* a binding site in the proximal *Il-21* promoter (26).

IL-21 is overproduced in many chronic inflammatory disorders, including inflammatory bowel diseases (IBD), rheumatoid arthritis, and SLE (9, 27, 28). In addition, blockade of IL-21 or IL21R has therapeutic effects on various murine models of inflammation and autoimmunity (11). Particulary, the role of IL-21 in IBD is well established. IL-21 modulates the activity of several cell types that contribute to tissue damage in IBD. With this regard, it is not surprising that mice lacking IL-21 are unable to upregulate Th17-associated molecules during experimental gut inflammation and are largely protected against chemically induced colitis (29, 30). IBD are chronic, relapsing inflammatory disorders of the gastrointestinal tract and involve multiple pathogenic factors including environmental changes, genetical predispositions, an abnormal gut microbiota, and a broadly dysregulated immune response (31). Current studies indicate that an active and dynamic interplay between immune and non-immune cells and gut microbiota play a major role in this pathologic process, and that cytokines, such as IL-21, are essential mediators of this crosstalk.

Previously, we have shown that that mice with a T cell-specific overexpression of CREMα show – beyond enhanced expression of IL-17a and decreased expression of IL-2 – also an enhanced IL-21 production both *in vitro* after T cell stimulation with anti CD3 and CD28 and *in vivo* in a contact dermatitis model (7). We, therefore, aimed to analyze molecular mechanisms of IL-21 transcription in these mice. Moreover, by a comparative analysis of CREMα and wild-type (WT) mice in an experimental colitis model, which is associated with T cellular IL-21 production, we wanted to study the consequences of CREMα-induced cytokine alterations.

## Materials and Methods

### Animals

Experiments were performed with age-matched FVB WT and CREMα tg FVB mice (7, 32) All mice were bred in our animal facility and kept under standardized conditions. Animals were housed in the same rooms under pathogen-free and helicobacter-free conditions. The study was approved by the regional government authorities and animal procedures were performed according to German legislation for animal protection. Permission for the projects has been granted by the Regierungspräsident/LANUV Nordrhein-Westfalen.

### *In Vitro* T Cell Stimulation

T cells were obtained from spleens of WT and CREMα tg mice by negative isolation using magnetic cell separation *via* CD4^+^ MACS Kits (Miltenyi, Germany) according to manufacturer’s instructions. Cells were cultured in RPMI medium supplemented with 1% penicillin/streptomycin and 10% inactivated fetal calf serum (Gibco Life Technologies, Germany) in individual wells of 96-well round-bottom microtiter plates. T cells were stimulated with anti-CD3 and anti-CD28 antibodies (both at 3 μg/ml; BD Bioscience, Germany) or with PMA (20 nM) and ionomycin (2 μM) (both Sigma-Aldrich, USA) as indicated.

### [Ca^2+^]*i* Measurement

Freshly isolated T lymphocytes (5 × 10^6^ cells/ml) were washed in phosphate-buffered saline (PBS), resuspended in RPMI containing 1% bovine serum albumin (BSA), and incubated for 40 min at 37°C with 1.3 μM Fluo-3-AM and 2.75 μM Fura-Red-AM (both from Invitrogen) in the presence of 0.02% Pluronic acid F-127 (Invitrogen). After incubation, cells were washed gently and resuspended in RPMI containing 0.1% BSA and incubated for 10 min at 37°C. Cells were then stimulated with anti-CD3 and anti-CD28, alternatively with PMA and ionomycin. Immediately after addition of the stimulus, Fluo-3/Fura-Red ratio as a measure of the released cytosolic Ca^2+^ concentration was monitored by FACS analysis. The quantification of the measurements was analyzed using FlowJo software (Tree Star, USA). For T lymphocytes stimulated with anti-CD3 and anti-CD28, [Ca^2+^]*i* was calculated from the equation: [Ca^2+^]*i* = Kd × Sf380/b380 (*R*–*R*min)/(*R*max–*R*), where Kd is 224 nm in the cytoplasmic environment; Sf380/b380 is the ratio of the intensities of the free and bound dye forms at 380 nm; *R* is the fluorescence ratio (340/380 nm) of the intracellular Fura-2; *R*max and *R*min are the maximal and minimal fluorescence ratios obtained by addition of Digitoxin and EGTA (final concentration 3 mmol/l), respectively.

### Chromatin Immunoprecipitation (ChIP)

The 3–4 × 10^6^ splenic CD3^+^ T-cells from CREMα tg and WT mice were stimulated for 45 min for CREM binding or 4 h for Histon 4 acetylation using Phorbol-12-myristat-13-acetat (30 nM) and ionomycin (1.5 µM) (both Sigma-Aldrich, USA) or left unstimulated. T-cells were cross-linked with 1% formaldehyde (Merck, Germany) for 5 min, washed, lysed, and sonicated. Supernatants were diluted in RIPA buffer. A proportion (10%) of the diluted supernatants was kept as input (PCR amplification of the total sample). For each immunoprecipitation reaction, an equal DNA amount was used. Samples were immunoprecipitated with 0.6–3 µg antibody (anti-CREM 2 µg; anti-NFAT 3 µg, both Abcam, UK; anti-Histon H4 Acetyl-K8 0.6 µg, Epitomics, USA) or with its isotype control (mouse monoclonal IgG1 or rabbit polyclonal IgG, both Abcam, UK) at 4°C overnight. Protein G Dynabeads (Novex by Life technologies, Germany) were incubated with the samples for 4 h at 4°C. Protein–DNA beads complexes were washed stringently with wash buffer (X-ChIP protocol from Abcam). Protein–DNA complexes were eluted in 1% SDS, 100 mM NaHCO_3_ for 15 min at 30°C, and reverse-cross-linked at 65°C overnight. For protein digestion, Proteinase K (20 mg/ml) was added to the samples and incubated for 2 h at 54°C. DNA was recovered using the Wizard kit (Promega, Germany) and subjected to PCR analysis on an ABI prism 7300 real-time PCR system. Primer sequences used for real-time PCR for the CREM-binding site or H4-acetylation of the *IL-21* promoter were 5′-GAAAACTGGAATTCACCCATGTCTCTCTT-3′ and 5′-AGAAGAGGCCAAGCCCTCCCATTG-3′. The immunoprecipitated DNA was calculated as relative to the respective input DNA. For H4-acetylation, the percent of input of the stimulated immunoprecipitated DNA was calculated as *n*-fold to the respective unstimulated DNA. Primer sequences used for the putative CREM-binding site of the CD3ζ *chain* promoter were 5′-CTGAATTGAGGCCAAGTTCAGA-3′ and 5′-AAGCATATATCT AAGTTGCTGTGTGG-3′. The immunoprecipitated CREMα tg DNA was calculated as *n*-fold to the respective WT DNA.

### Reporter-ChIP (R-ChIP)

Reporter ChIP technique enables the study of the *in vivo* binding of transcription factors to a specific binding site on a promoter of a reporter construct (33). To analyze CREM binding to the mutated or not-mutated *Il-21-luc* promoter, 5 Mio EL-4 culture cells were spread in six-well plates in 3 ml RPMI + 5% FCS (without P/S) and left to rest for 2 h at 37°C. EL-4 cells were transfected with 6 µg *Il-21-luc* promoter plasmid *Il-21-luc* promoter mutated plasmid and K2 transfection reagent overnight. Cells were split the next day and incubated for 4 h with PMA (20 nM) and ionomycin (2 µM) or left unstimulated. After 4 h, cells were harvested for R-ChIP and analyzed for CREM binding as described above.

### Plasmid Construction and Mutagenesis

The 267-bb IL-21 promoter region (25) was amplified by PCR from genomic DNA of FVB WT mice using Q5 Hot Start High-Fidelity DNA Polymerase (NEB, USA) and cloned into *Kpn*I and *Bgl*II sites of the pGL3 plasmid (Promega, USA) to generate the *Il-21-luc* construct. Oligonucleotides used for genomic PCR were 5′-GCAGATCTGATGACAGGGCCTTGGTCTG-3′ and 5′-ACGGTACCTCAGACAAA CCAGGTGAGGTG-3′. Mutagenesis of the *half-CRE* site within the *Il-21-luc* plasmid was performed with the Q5 Site-Directed Mutagenesis Kit (NEB, USA) according to manufacturer’s instructions. Oligonucleotides used for mutagenesis of the *half-CRE* site cagt into ccgg were 5′-CTTCAACCTGccggTGCACAGGTTGTCG-3′ and 5′-TGAGAACCAGACCAAGGTG-3′.

### Luciferase Reporter Gene Assays

EL-4 cells were transfected with the K2 Transfection System (Biontex, Germany) by using 1 × 10^6^ cells and a total of 1.5 µg of indicated reporter plasmids and expression plasmids or pcDNA according to manufacturer’s instructions. Whenever an effector:reporter co-transfection was performed, the molar ratio between the two was 3:1. Before transfection cells were incubated with 20 µl/ml Multiplayer (K2 Transfection System) for 2 h at 37°C. All cells were co-transfected with 0.5 µg of *Renilla* luciferase promoter plasmid (pRL-TK) as a normalization control for transfection efficiency. Cells were left to rest over night before luciferase activity was measured by using the Dual-Glo Luciferase Assay System (Promega, USA). For experiments with cyclosporin A (CSA), transfected cells were incubated with or without 1 µg/ml CSA for 1 h and stimulated with PMA (20 nM) and ionomycin (2µM) for 5 h before luciferase activity was measured. Expression plasmid NFATc2 was a kind gift from Tobias Bopp (University of Mainz, Institute of Immunology) and the expression plasmid CREMα was a kind gift from Dr. P. Sassone-Corsi (University of California, Irvine, CA, USA).

### Induction of Colitis

Acute dextran sodium sulfate (DSS) colitis was induced by giving mice 3.5% (w/v) DSS (molecular mass, 36–50 kDa; MP Biomedicals, USA) in drinking water *ad libitum* for 6 days. Body weight and fecal status were followed daily from the time of colitis induction. Mice were killed on day 6 when some of the animals had lost more than 15% of their initial body weight. Spleen, mesenterial, and peripheral lymph nodes were harvested for further analysis. The biggest part of the colon was fixed in formalin, and the other small part was washed with PBS and frozen in RNA later (Qiagen, Germany) for mRNA analysis.

### Immunization

For T cell-dependent immunization, groups of age-matched mice were immunized i.p. with 100 µg 4-hydroxy-3-nitrophenylacetyl chicken gamma globulin (NP-CGG) (Biosearch Technologies, USA) in Imject Alum (Thermoscientific, USA). Spleens were harvested 7 days later.

### Histological Scoring

Four micrometers of paraffin sections from the fixed colon were serially cut, mounted onto glass slides, and deparaffinized. The colon sections were stained with hematoxylin and eosin (H&E) by the Core Facility (IZKF) of the RWTH Aachen University. Blinded histological scoring was performed using a standard microscope, based on The Jackson Laboratory Scoring (TJL) method, as described previously (34, 35). Each colon section was scored for the four general criteria: severity, degree of hyperplasia, degree of ulceration, if present, and percentage of area involved. A subjective range of 1–3 (1 = mild, 2 = moderate, 3 = severe) was used for the first three categories. Severity: focally small or widely separated multifocal areas of inflammation limited to the lamina propria were graded as mild lesions (1). Multifocal or locally extensive areas of inflammation extending to the submucosa were graded as moderate lesions (2). If the inflammation extended to all layers of the intestinal wall or the entire intestinal epithelium was destroyed, lesions were graded as severe (3). Hyperplasia: mild hyperplasia consisted of morphologically normal lining epithelium that was two or more times thicker (length of crypts) than adjacent or control mucosa. Moderate hyperplasia was characterized by the lining epithelium being two or three times normal thickness, cells were hyperchromatic, numbers of goblet cells were decreased, and scattered individual crypts developed an arborizing pattern. Severe hyperplastic regions exhibited markedly thickened epithelium (four or more times normal), marked hyperchromasia of cells, few to no goblet cells, a high mitotic index of cells within the crypts, and numerous crypts with arborizing pattern. Ulceration was graded as: 0 = no ulcer, 1 = 1–2 ulcers (involving up to a total of 20 crypts), 2 = 1–4 ulcers (involving a total of 20–40 crypts), and 3 = any ulcers that exceed the previous. A 10% scale was used to estimate the area involved in the inflammatory process: 0 = 0%, 1 = 10–30%, 2 = 40–70%, 3 = >70%.

### Flow Cytometric Analysis

For surface staining, single-cell suspensions were isolated from spleens and mesenterial lymph nodes (mLNs) after 6 days of colitis induction and stained with anti CD4-FITC (eBioscience, USA) and anti CD4-APC (BD Bioscience, Germany). For measurement of intracellular cytokines, cells were treated with P/I and GolgiPlug (BD) for 5 h and fixed and permeabilized with FoxP3 staining buffer set (eBioscience) following the manufacturers’s instructions. Intracellular cytokines were stained with anti-IL-21-Alexa 647 (eBioscience) and anti IL-17-Alexa 488 (BD) antibodies. Intracellular staining of CD3ζ was performed using Cytofix/Cytoperm Kit (BD) and anti CD3ζ-PE antibody (BD) according to the manufacturer’s instructions. Samples were analyzed using a FACS Canto II (BD) and data analyzed using FCS Express (*De Novo* Software) software.

### RNA Isolation and Real-time PCR

Total RNA from cells of mLN, inguinal lymph nodes, and colon tissue after DSS treatment was isolated using RNeasy Mini Kit (Qiagen, Germany). cDNA was then generated from 700 ng total RNA using RevertAid H Minus First Strand cDNA Synthesis Kit (Thermo Fisher Scientific, USA) according to the manufacturer’s instructions. Real-time PCR was performed using SYBR Green PCR kit (Eurogentec, Germany) and data were acquired with the ABI prism 7300 real-time PCR system (Applied Biosystems by Life Technologies, Germany). Each measurement was set up in duplicates, and two independent experiments were performed. After normalization to endogenous housekeeping control gene β-Actin for mice, the relative expression was calculated. The following primers were used: 5′-AAGATTCCTGAGGATCCGAGAAG-3′ and 5′-GCATTCGTGAGCGTCTATAGTGTC-3′ for IL-21 [adapted from Ref. (36)] 5′-AGCTGGACCACCACATGAATT-3′ and 5′-CCACACCCACCAGCATCTTC-3′ for IL-17; 5′-CCAGTGTGAAGATGGTTGTGACC-3′ and 5′-GGTGCTTATAAAAAGCCAGACCTTG-3′ for IL-23; 5′-TGCCAAGTTTGAGGTCAACAACCCA-3′ and 5′-CCCACCCCGAATCAGCAGCG-3′ for IFN-γ; 5′-ACTATTGGCAACGAGCGGTTC-3′ and 5′-TTACGGATGTCAACGTCACACTTC-3′ for β-Actin. mRNA expression levels of IL-21 in mLNs were compared to IL-21 mRNA expression levels in peripheral lymph nodes (*n*-fold). mRNA expression levels of IL-21, IL-17, IL-23, and IFN-γ in colon tissue of CREMα tg mice were compared to the their respective mRNA expression levels of their untreated WT littermates (*n*-fold).

mRNA expression of the CD3ζ chain was determined in CD4 positive T cells extracted by MACS isolation with the following primers: 5′-ATTGTAGAGCTGGTTGGGGTCCTG-3′ and 5′-CATCCTCCACGTGCGGTTCC-3′. *n*-fold of CD3ζ chain mRNA expression in CREMα tg mice was calculated to the CD3ζ chain mRNA expression of their WT littermates.

Expression of cMAF, Bcl-6, and IRF4 was determined in isolated T cells, which were stimulated with P/I for 6 h or left unstimulated. The following primers were used: 5′-GCC CAA CAA GCT AGA AAG-3′ and 5′-TCT CTG AGG GTC TGG AAA CT-3′ for IRF4; 5′-AGG GAC CTG TTC ACG AGA TTA TTG-3′ and GTC CCC GAC CAA GCT CAG T for Bcl-6; 5′-AGCAGTTGGTGACCATGTCG-3′ and 5′-TGGAGATCTCCTGCTTGAGG-3′ for cMAF.

### Statistical Analysis

All data are presented as mean ± SEM. Differences between two groups were evaluated using unpaired or paired, when necessary, two-sided (or one-sided, where specifically indicated) Student’s *t*-test when data were normally distributed. Otherwise, a non-parametric Mann–Whitney test was performed. All statistical analysis and subsequent graphics generations were performed using GraphPad Prism version 5.0 (GraphPad Software, USA). A *p*-Value <0.05 was considered significant.

## Results

### CREMα Enhances IL-21 Production in T Cells

**Figure 1:**
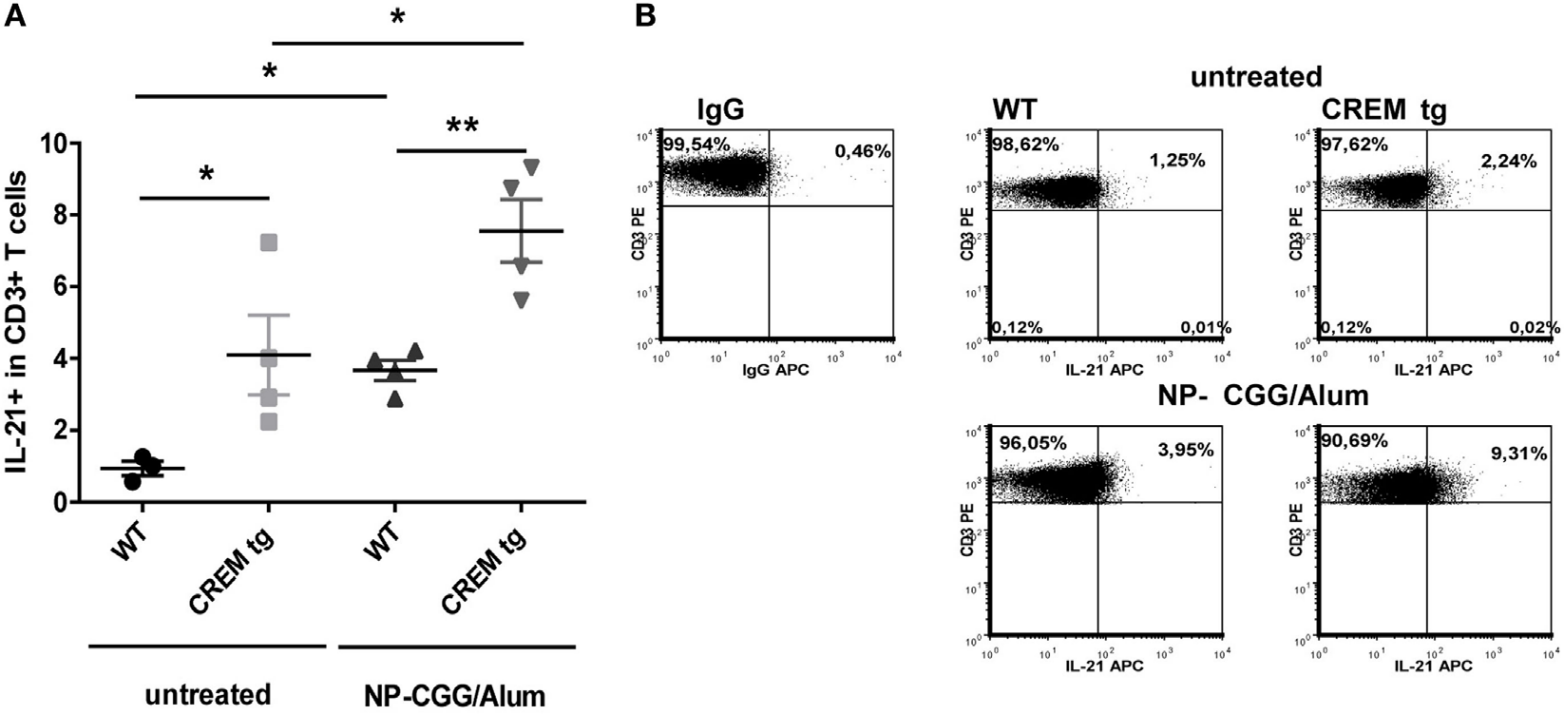
**cAMP-responsive element modulator alpha overexpressing T cells produce enhanced levels of IL-21**. **(A)** Percentages of IL-21^+^ cells within CD3^+^ T cells in spleens of untreated mice or mice after immunization with NP-CGG/Alum. Data show mean percentage ± SEM, *p < 0.05. **(B)** Representative flow cytometric dot plots of IL-21 expressing CD3^+^ T-cells in a CD3^+^-Gate.

Previously, we have shown that mice with a T cell-specific overexpression of CREMα show an enhanced IL-21 production both *in vitro* after T cell stimulation with anti-CD3 and anti-CD28 and *in vivo* in a contact dermatitis model (7). To further confirm that CREMα regulates IL-21 expression, we immunized WT and CREMα transgenic (CREMα tg) mice with the T cell-dependent antigen NP-CGG. Untreated CREMα tg mice already showed a higher frequency of IL-21 producing cells within the CD4^+^ T cell population (Figures 1A,B). Seven days after immunization IL-21 production was enhanced in WT and CREMα tg mice compared to untreated mice but to higher extent in CREMα tg mice (Figures 1A,B). These data confirm our previously published observation that CREMα enhances IL-21 expression both *in vitro* and *in vivo*.

### CREMα Suppresses CD3ζ Chain Expression Resulting in Enhanced Calcium Influx

**Figure 2:**
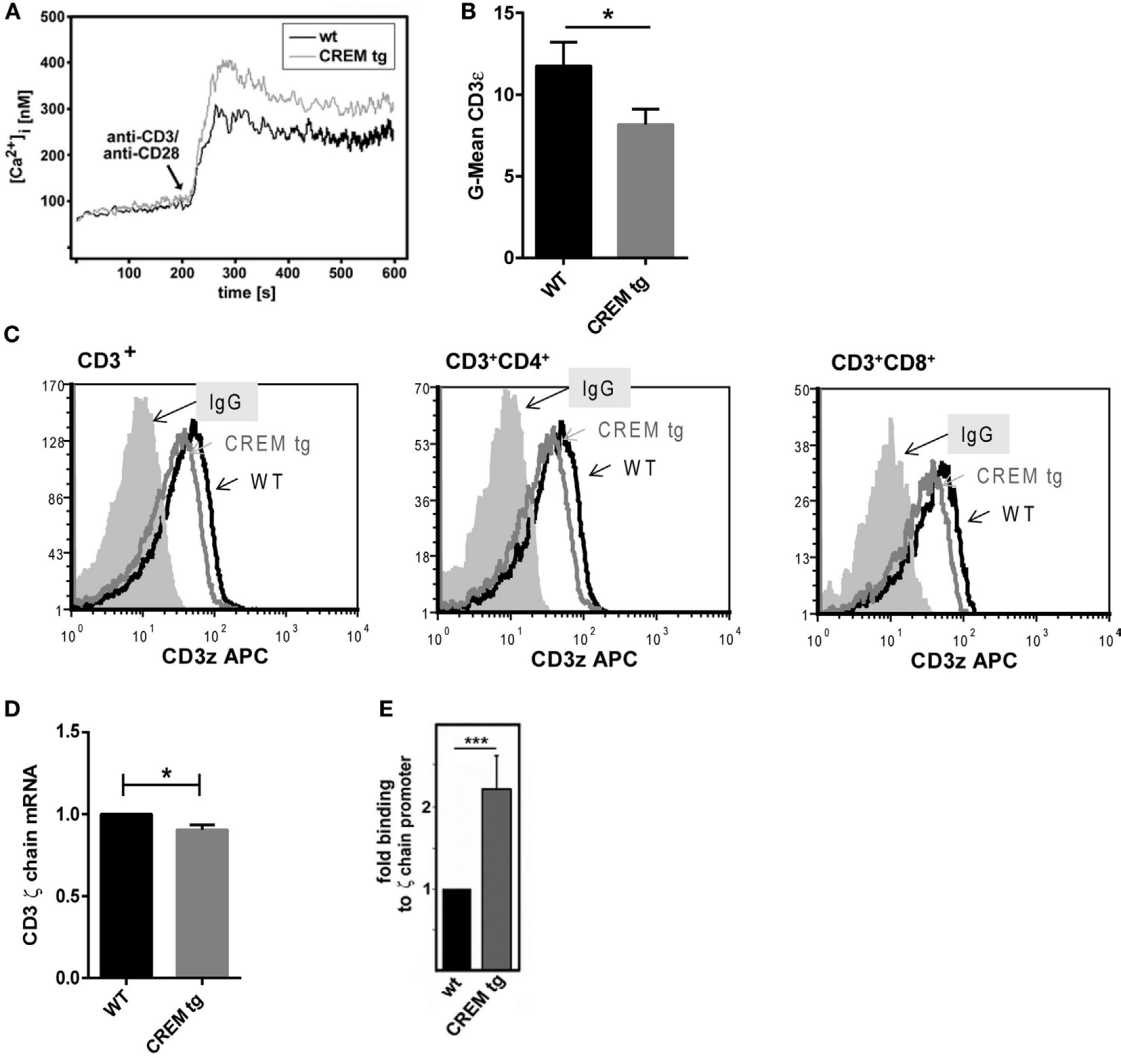
**cAMP-responsive element modulator alpha (CREMα) represses expression of CD3ζ chain, which leads to enhanced calcium influx**. **(A)** Pan-T cells were isolated *via* MACS isolation from CD2-CREMα TG mice and appropriate wild-type (WT) mice. Ca^2+^ influx was measured immediately after stimulation with anti-CD3 and anti-CD28. The picture shows a typical experiment (*n* = 4). **(B)** CD3ζ chain expression was measured in CD3^+^ T cells of CREMα transgenic and WT mice by flow cytometry. **(C)** The picture shows a typical histogram of CD3ζ chain expression in CD3^+^ (left), CD3^+^CD4^+^ (middle), and CD3^+^CD8^+^ T cells (right). **(D)** CD4 positive T cells were extracted by MACS isolation and mRNA expression of the CD3ζ chain was determined (*n* = 3, *p < 0.05). **(E)** Binding of CREM to the CD3ζ promoter was determined by chromatin immunoprecipitation assay in unstimulated splenocytes of CREMα transgenic and WT mice (*n* = 4, ***p < 0.005).

IL-21 expression depends on increased intracellular calcium (Ca^2+^) levels which activate NFAT that in turn activates *Il21* gene transcription (23). Accordingly, we evaluated Ca^2+^ responses in both WT and CREMα tg T cells. Compared to WT T cells, we noted an enhanced-free intracytoplasmic Ca^2+^ response in CREMα tg T cells that had been stimulated with anti-CD3/CD28 antibodies (Figure 2A). No difference was found in T cells that had been stimulated with PMA and ionomycin (Figure S1A in Supplementary Material) suggesting that this difference is related to alterations in the CD3 complex. Interestingly, both enhanced Ca^2+^ signaling and NFAT activity have been reported in T cells of SLE patients (37, 38). Enhanced calcium influx is observed in SLE T cells as well and was linked to a decreased expression of the CD3ζ chain (38). In detail, reduced CD3ζ chain expression in human SLE T cells results in enhanced calcium signaling and vice versa transfection of SLE T cells with the ζ chain construct corrected the heightened calcium response in SLE T cells (39). Previously, we have shown that CREMα binds to the human CD3ζ *chain* promoter and represses its activity (40). Accordingly, we checked expression of the CD3ζ chain and found it decreased in both CD4^+^ and CD8^+^ T cells of CREMα tg animals compared to WT animals on protein (Figures 2B,C) as well as on mRNA level (Figure 2D), whereas the overall expression of CD3 was not altered (Figure S1B in Supplementary Material). Moreover, we screened the murine *CD3*ζ *chain* promoter for CRE sites and identified a putative-binding site of CREMα within this promoter. Congruent to the CRE site within the human CD3 promoter, which we already showed to be regulated by CREMα, the identified CRE site in the murine *CD3*ζ *chain* promoter is located about 390 bp upstream of the transcription start. ChIP experiments using an anti-CREM antibody confirmed an up to twofold binding of CREMα to this promoter (Figure 2E). Thus, the transgenic overexpression of CREMα in T cells probably results in a decreased CD3ζ chain expression, thereby causing an enhanced Ca^2+^ influx, which may be responsible for the enhanced *Il-21* transcription.

### CREM Binds to and Activates the Proximal IL-21 Promoter Region

**Figure 3:**
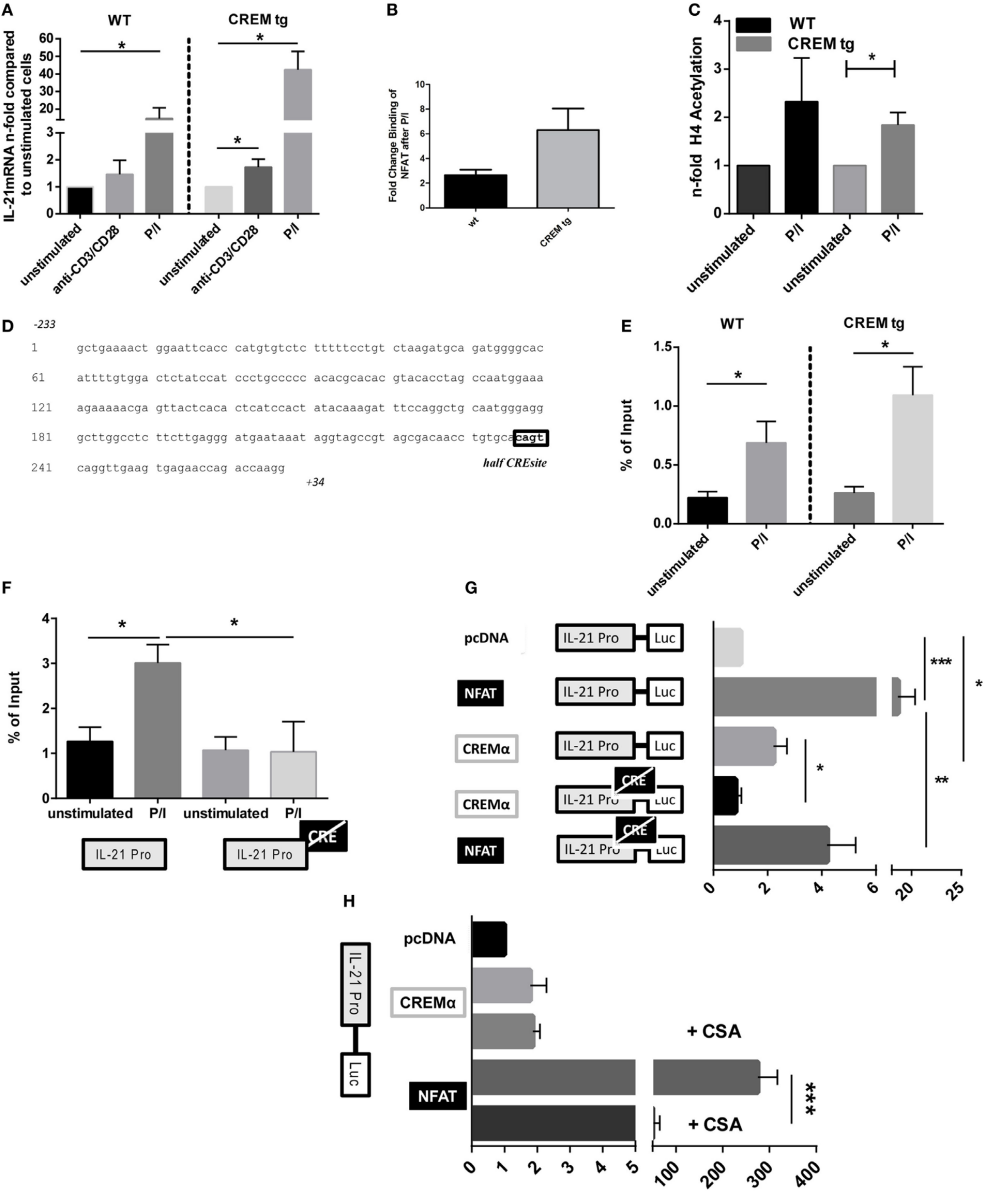
**cAMP-responsive element modulator alpha (CREMα) binds to and directly activates the proximal IL-21 promoter region**. **(A)** Splenic CD4^+^ T cells from CD2-CREMα transgenic (tg) mice and their wild-type (WT) littermates were stimulated with anti-CD3/CD28 antibodies or P/I for 12 h, mRNA was isolated from these cells, and measured for IL-21 cytokine expression compared to appropriate unstimulated cells (*n* = 4, *p < 0.05). **(B)** Splenic CD3^+^ T cells from CD2-CREMα tg mice and their WT littermates were stimulated with P/I for 4 h and chromatin immunoprecipitation (ChIP) assay was performed with anti-NFAT (*n* = 3). **(C)** Splenic CD3^+^ T cells from CD2-CREMα tg mice and their WT littermates were stimulated with P/I for 4 h, and ChIP assay was performed with anti-H4 Acetyl-K8 antibody (*n* = 4, one-sided, paired *t*-test *p < 0.05). **(D)** Genomic sequence of the murine IL-21 promoter region with the indicated CREM-binding *half CRE site cagt* within the promoter. **(E)** CD3^+^ T-cells were isolated from spleens of CREMα tg mice and appropriate WT mice and stimulated with P/I for 45 min or left unstimulated. Binding of CREM to the IL-21 promoter was determined by ChIP assays (*n* = 6, *p < 0.05). **(F)** EL-4 cells were transfected with either *IL-21-luc* or *IL-21-luc* with a mutated *half-CRE* site and either an empty control vector (pcDNA) or a plasmid-expressing CREMα. CREM binding was assessed by reporter-ChIP (*n* = 4, *p < 0.05). **(G)** EL-4 cells were transfected with either *IL-21-luc* or *IL-21-luc* with a mutated *half-CRE* site and either an empty control vector (pcDNA) alone, or plasmid expressing NFATc2 or CREMα. Cells were left to rest over night before luciferase activity was measured. Activity is expressed as the fold induction in luciferase activity relative to IL-21 pro-luc reporter activity cotransfected with pcDNA and is adjusted for transfection efficiency with a co-transfected *Renilla* luciferase promoter plasmid (pRL-TK) (*n* = 4, *p < 0.05, ***p < 0.005). **(H)** EL-4 cells were transfected with *IL-21-luc* and either an empty control vector (pcDNA), or plasmid-expressing NFATc2 or CREMα. Cells were stimulated with P/I in the presence or absence of cyclosporin A and luciferase activity was measured (*n* = 3, ***p < 0.001).

We next stimulated splenic T cells from WT and CREMα tg mice *in vitro* either with anti-CD3/CD28 antibodies or with Phorbol-12-myristat-13-acetate and ionomycin (P/I) and analyzed *Il-21* mRNA expression (Figure 3A). As expected, stimulation with anti-CD3/CD28 antibodies significantly upregulated *Il-21* expression of CREMα tg T cells. However, stimulation with P/I resulted in a much higher and significant induction of *Il-21* mRNA expression in both WT and CREMα tg T cells. This implies that the IL-21 expression in CREMα tg T cells might in part be due to a low CD3ζ expression, but that pathways downstream of the TCR signaling complex must also be involved, since P/I acts independently from the CD3ζ complex. However, NFAT binding was insignificantly enhanced in CREMα transgenic T cells after stimulation (Figure 3B), which on the other hand suggests that calcium/NFATc2-dependent mechanism at least partially influence IL-21 transcription in CREMα tg T cells. Stimulation of WT and CREMα tg T cells with P/I resulted in enhanced acetylation on histone 4 of the IL-21 promoter in comparable manner (Figure 3C), which means that CREMα does not change acetylation of histone 4. We, furthermore, did not detect differential expression of c-Maf, IRF4, and Bcl-6, which are known to be associated with IL-21 induction (Figure S2 in Supplementary Material) (41–43). We, therefore, assumed that CREMα might also directly regulate IL-21 expression itself. To determine whether *Il-21* was a direct target of the transcription factor CREMα, the *Il-21* promoter sequence was screened for potential *CRE*-binding sites. Mehta et al. already identified and characterized a murine *IL-21* proximal promoter region, which is located immediately 5′of the first coding exon. This region is highly conserved between men and mice and activates IL-21 transcription (25). This region also contains a binding site matching a consensus half-site for CREM (half *CRE*-site) (Figure 3D). We next sought to confirm CREM binding to the *Il-21* promoter *in vivo* using ChIP assays with murine splenic CD3^+^ T cells. CREM binding to the proximal *IL-21* promoter region was enhanced in WT T cells as well as in CREMα tg T cells after stimulation with P/I; however, we did not find a significant difference in binding between CREMα tg T cells and WT T cells (Figure 3E). To identify if the site is indeed important for binding of CREM, we used the R-ChIP technique, which we recently established and which enables the study of the *in vivo* binding of transcription factors to a specific binding site on a promoter of a reporter construct. Using this technique, we could show a specific binding of CREM to this *CRE* site. Transfection of EL-4 cells with a *IL-21 promoter* construct spanning 267 bp of the *Il-21* promoter (*Il-21-luc*) containing a mutated *CRE* site almost abolished CREM binding after stimulation compared to the non-mutated *IL-21 promoter* plasmid (Figure 3F). To further test the specific function of the CREM-binding site in the *Il-21* promoter, we used the abovementioned luciferase construct (*Il-21-luc*). We transfected EL-4 T cells with the *Il-21-luc* reporter plasmid including co-transfection of expression plasmids for CREMα or NFATc2 and measured luciferase activity compared to an empty control vector (pcDNA) (Figure 3F). NFATc2 plasmid served as a control in our setting as activation of IL-21 transcription by NFATc2 is well known (23, 25). Consequently, NFATc2-transfected cells revealed an about 20-fold increased *IL-21-luc* activity compared to cells transfected with pcDNA. Transfection with *Il-21-luc* and an expression vector for CREMα led to an about twofold-enhanced *Il-21-luc* activity compared to co-transfection with pcDNA suggesting that CREMα is at least partially responsible for the activity of the *Il-21* promoter. The mutagenesis of the half-*CRE* site prevented upregulation of *Il-21-luc* activity in the presence of exogenous CREMα (Figure 3G), which is in accordance with the reduced binding shown by R-ChIP in Figure 3F. However, promoter activity was also reduced in the presence of exogenous NFATc2. This somehow unexpected finding probably results from chromatin remodeling or CREM/NFAT interactions suggesting crucial importance of this site for the whole promoter activity. To further dissect the direct and Ca^2+^-mediated effects of CREMα on IL-21 expression, we evaluated the luciferase activity of cells transfected with *IL-21-luc* and CREM or NFATc2 plasmids in the presence of CSA. CSA is an immunosuppressive drug and inhibits calcineurin that, in turn, results in the blockade of the transcription factor NFATc2 (44). As a consequence, NFATc2-mediated *Il-21-luc* activity was abrogated in stimulated cells in the presence of CSA. However, CREM-mediated *Il-21-luc* activity was not altered by CSA (Figure 3H). These data show that the direct effect of CREMα on *IL21 promoter* activity is mediated independently of NFATc2. We conclude from these experiments that CREMα directly binds to the proximal *Il-21* promoter and thereby activates IL-21 transcription.

### CREMα Transgenic Mice Display Enhanced Disease Activity in DSS-Induced Colitis

**Figure 4:**
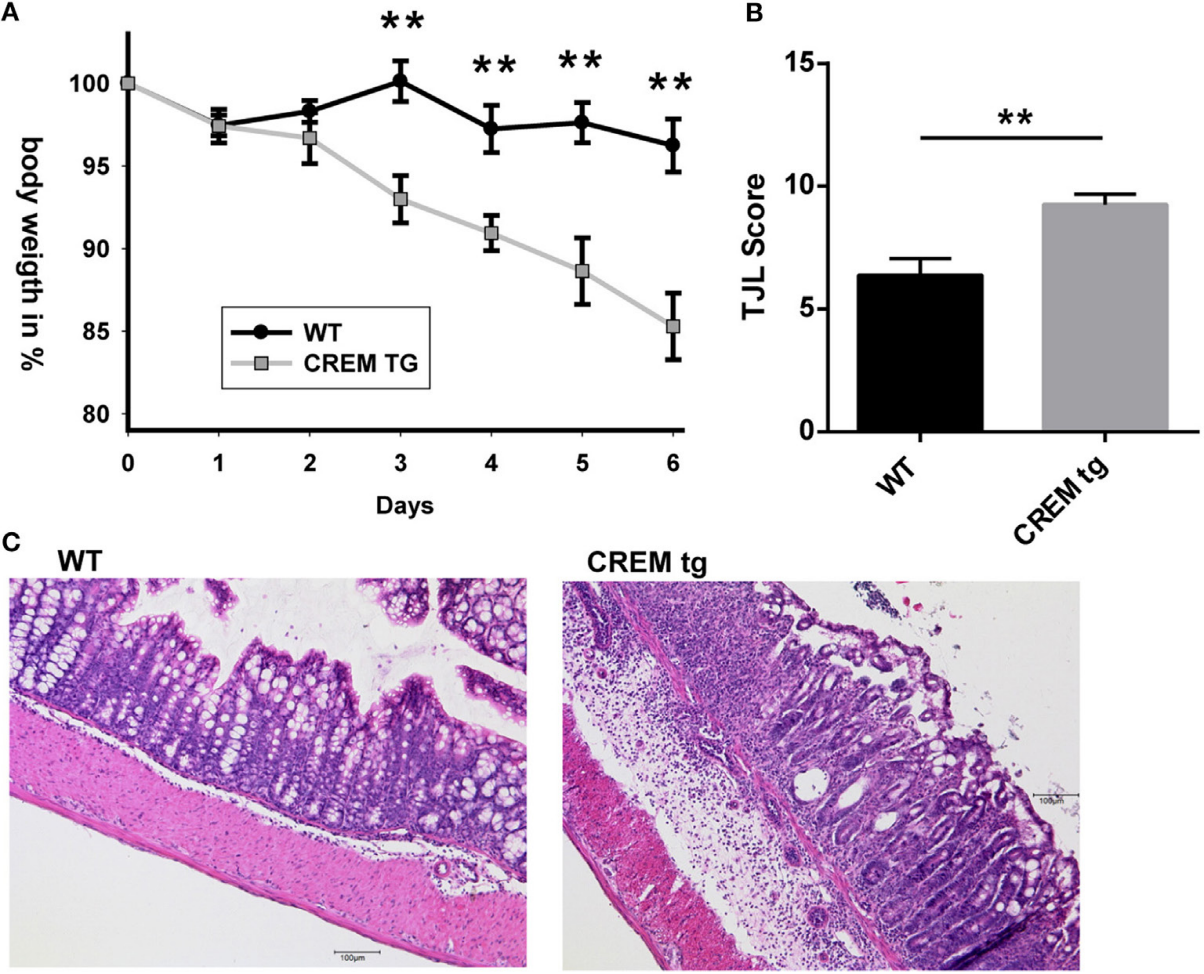
**cAMP-responsive element modulator alpha (CREMα) transgenic mice display enhanced disease activity in dextran sodium sulfate (DSS)-induced acute colitis**. **(A)** Percentage of body weight loss of CD2-CREMα transgenic (tg) and wild-type (WT) mice measured daily for a period of 6 days after colitis induction with DSS (*n* = 12 animals each, **p < 0.01). **(B)** Results of histological scoring of colon sections from CD2-CREMα tg and WT mice on day 6 after DSS treatment (*n* = 12, **p < 0.01). **(C)** Representative photomicrographs of hematoxylin and eosin-stained colon sections from CD2-CREMα tg (right) and WT (left) mice at day 6 of acute DSS colitis imaged using a 10× objective (scale bar = 100 µm).

IL-21 supports generation and stabilization of pathogenic Th17 cells (13, 29), which might be one of the mechanisms by which IL-21 expands and sustains ongoing mucosal inflammation. IL-17 expression in CREMα tg mice is probably to some extent regulated by IL-21, as was shown by blockade experiments with IL-21RFc, which prevented RORyt and IL-17 expression *in vitro* (7). We next asked how CREMα tg T cells affect disease activity and cytokine release in a murine colitis model. We, therefore, used an established murine model of colitis, in which colitis is induced by oral administration of DSS. Wild-type and CREMα tg mice were given 3.5% DSS in their drinking water. While CREMα tg mice suffered from a progressive weight loss, WT mice hardly lost weight (Figure 4A). Histological changes following DSS treatment were detectable in both groups, but the extent of tissue damage was greater in CREMα tg mice compared to WT mice (Figures 4B,C). We conclude from this that overexpression of CREMα in T cells enhances inflammation in DSS-induced colitis.

### Colitis in CREMα tg Mice Is Accompanied by Higher Local IL-21 Expression

**Figure 5:**
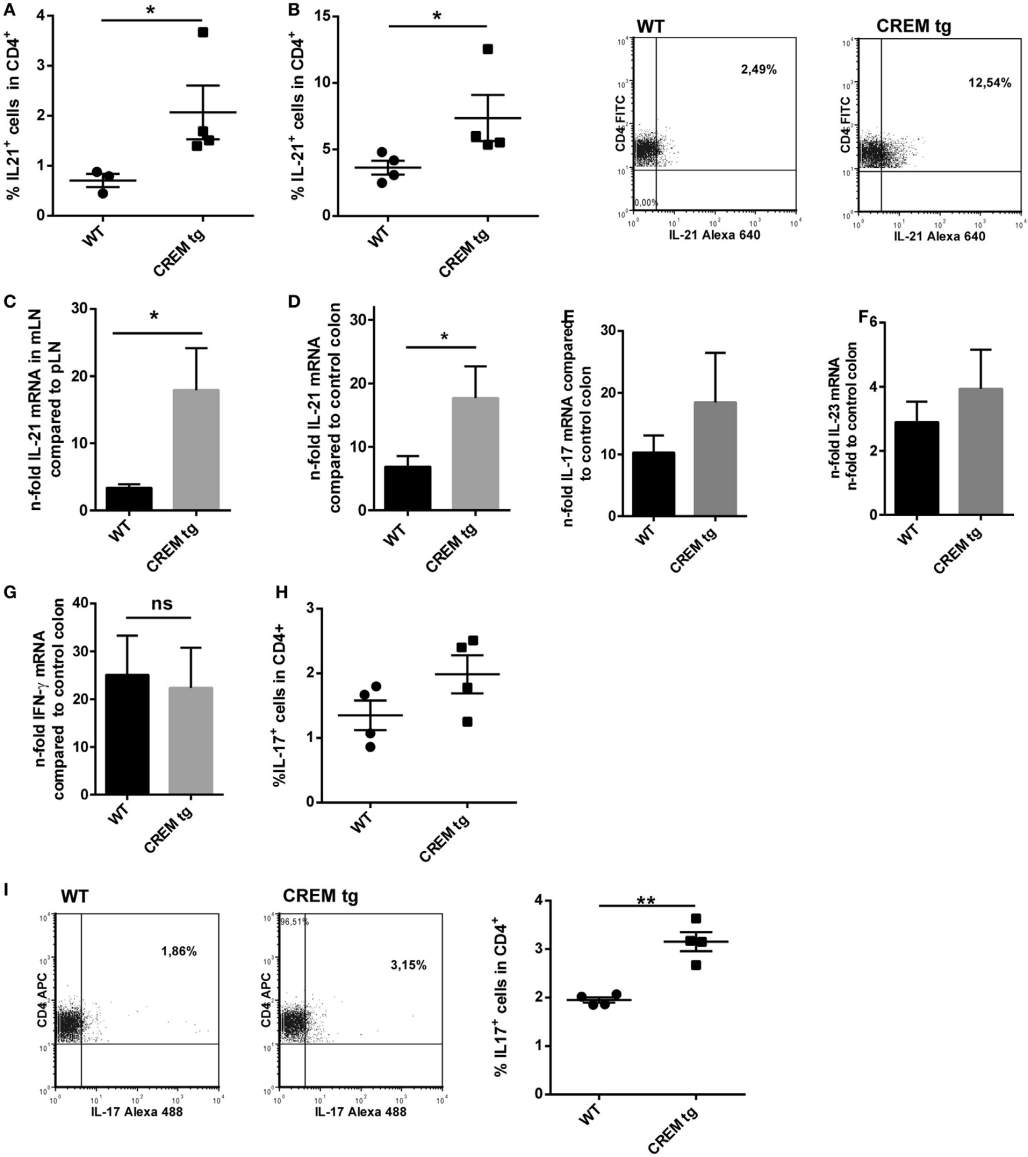
**Colitis in cAMP-responsive element modulator alpha (CREMα) transgenic (tg) mice is accompanied by higher local IL-21 expression**. **(A)** Percentages of IL-21 expressing CD4^+^ T-cells in mesenterial lymph node (mLN) and **(B)** in spleen of age-matched CREMα tg mice and appropriate wild-type (WT) mice after 6 days of colitis induction measured by flow cytometry. Data show mean percentage ± SEM of IL-21 expressing CD4^+^ T-cells in at least four mice per group (*p < 0.05). Representative flow cytometric dot plots of IL-21-expressing CD4^+^ T-cells in a CD4^+^-gate on the right. **(C)** mRNA levels of IL-21 in mLN cells (*n*-fold compared to IL-21 mRNA in peripheral LN) of CREMα tg mice compared to their WT littermates after 6 days of colitis induction measured by real-time quantitative PCR (*n* = 4, *p < 0.05). **(D)** mRNA levels of IL-21 in colon tissue of CREMα tg mice and appropriate WT mice after 6 days of dextran sodium sulfate (DSS) treatment compared to their untreated WT controls (*n*-fold) measured by real-time quantitative PCR (*n* = 4, *p < 0.05). **(E)** mRNA levels of IL-17, **(F)** IL-23, and **(G)** IFN-γ in colon tissue of CREMα tg mice and appropriate WT mice after 6 days of DSS treatment compared to their untreated WT controls (*n*-fold) measured by real-time quantitative PCR (*n* = 4–5). **(H)** Percentages of IL-17-expressing CD4^+^ T-cells in mLN and **(I)** in spleen of age-matched CREMα tg mice and appropriate WT mice after 6 days of colitis induction measured by flow cytometry. Data show mean percentage ± SEM of IL-17-expressing CD4^+^ T-cells in at least four mice per group (**p < 0.01). Representative flow cytometric dot plots of IL-17-expressing CD4^+^ T-cells in a CD4^+^-gate on the right.

To further analyze if higher disease activity also involves higher expression of IL-21 and IL-17, we analyzed IL-21 protein expression by flow cytometry in spleen and mLNs of colitic mice. CREMα tg mice showed enhanced T cellular IL-21 production in mLN (Figure 5A) and spleen (Figure 5B) compared to WT animals. Additionally, mRNA levels of *Il-21* were also enhanced in mLN (Figure 5C) of CREMα tg mice compared to WT mice and colon tissue also showed enhanced *Il-21* mRNA expression (Figure 5D), while there was an insignificant tendency toward enhanced IL-17 transcription (Figure 5E). IL-23 (Figure 5F) and IFN-γ expression was not altered between both groups neither on mRNA (Figure 5G) nor on protein level (not shown). IL-17^+^ T cells were not enhanced in mLN (Figure 5H) from CREMα tg mice, but significantly enhanced in spleens (Figure 5I). These data proof enhanced production of the inflammatory cytokine IL-21 in CREMα tg colitic mice, which potentially accounts for accelerated disease activity.

## Discussion

**Figure 6:**
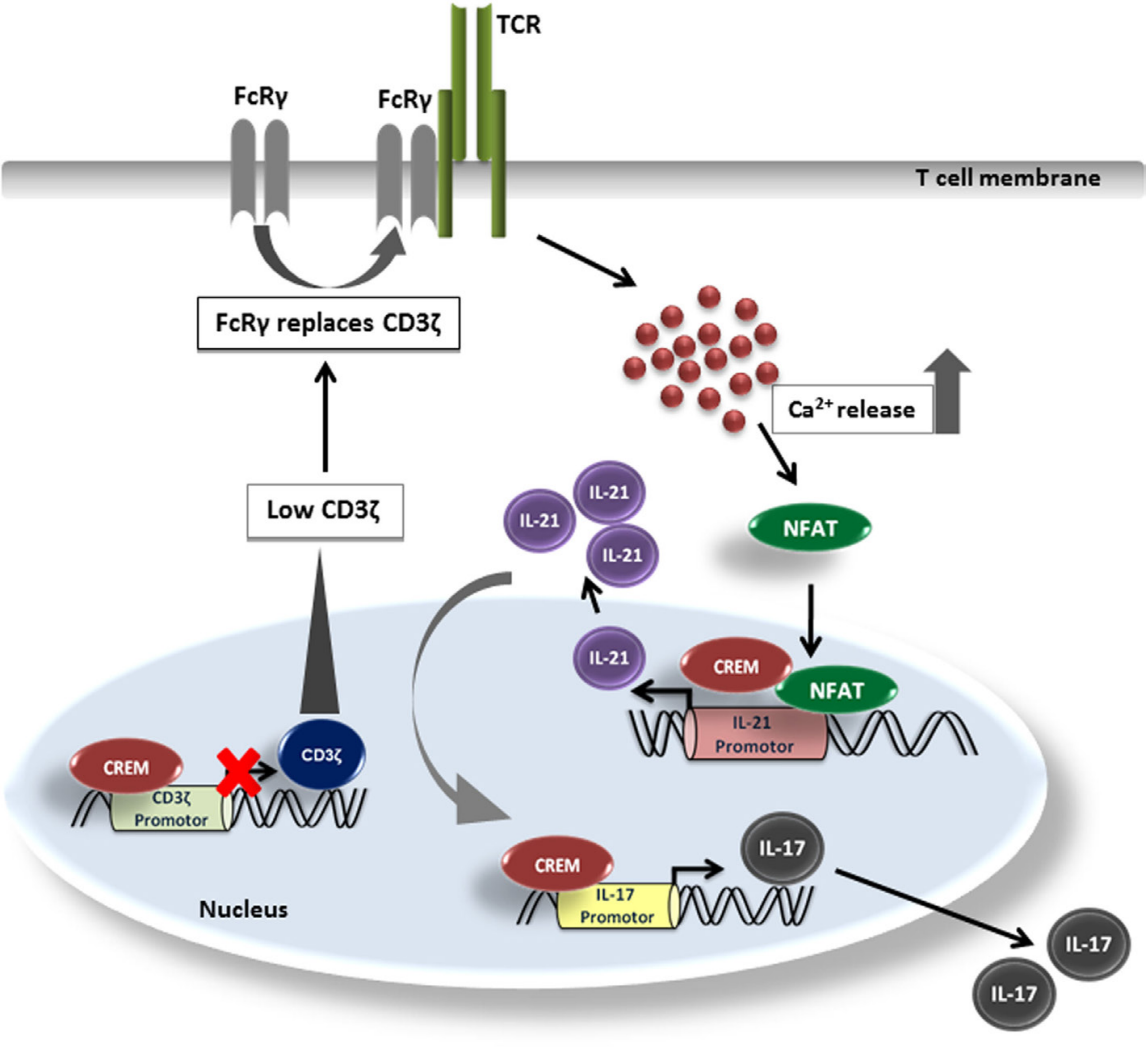
**Model of IL-21 induction by cAMP-responsive element modulator alpha (CREMα)**. CREMα represses CD3ζ chain expression. CD3ζ chain, which normally transduces TCR signals into the cell, can be replaced by FcRγ. FcRγ signals much stronger than the CD3ζ chain. Therefore, higher amounts of Ca^2+^ are released, which lead to an enhanced binding of NFATc2 to the IL-21 promoter and subsequently induces IL-21 expression. At the same time, CREMα also binds to the *half-CRE* site in the IL-21 promoter, which is only part due to the low CD3ζ expression as pathways downstream of the TCR complex signaling must be also involved. CREMα likewise binds to the IL-17 promoter and increases IL-17 expression while IL-21, to some extent, supports generation and stabilization of the IL-17 expression.

In this work, we describe the role of CREMα for the transcriptional activity of the *Il-21* promoter. We analyzed two mechanisms that might account for the enhanced IL-21 production in CREMα tg mice. First, CREMα binds to the CD3ζ chain promoter resulting in a downregulation of CD3ζ chain expression. This mechanism has been observed in SLE patients before which display enhanced T cellular levels of CREMα (40). Downregulation of the CD3ζ complex results in altered lipid raft formation, lower excitation threshold, and enhanced Ca^2+^ signaling (45). In analogy to the human disease, we also found enhanced Ca^2+^ signaling in T cells of the CREMα tg mice. Ca^2+^ is central for IL-21 transcription, since it activates NFAT, which is the most important transcription factor for activation of the *IL-21* promoter (23). Nevertheless, apart from this we also found a striking upregulation of *Il-21* transcription in CREMα tg T cells by P/I stimulation, which suggest a mechanism independent from the T cell receptor. We thus screened the murine *Il-21* promoter and identified a *CRE*-halfside, which is crucial for the whole promoter activity and is able to bind CREMα and most probably accounts for transcriptional activation mediated by this transcription factor. This is supported by two experiments; first, R-ChIP shows abrogated binding of CREM on the mutated CRE-site, second, calcineurin treatment does not affect activity of CREM on the IL-21 promoter, which both suggest NFAT-independent effects. Thus, in analogy to the murine and human *IL-17* promoter, CREMα also displays activator function for this proinflammatory cytokine (Figure 6). This could have several implications. We have shown before that blockade of IL-21 by an IL21RFc fragment downregulates IL-17 production in the CREMα tg T cells *in vitro* (7), which underpins the importance of CREM-mediated IL-21 transcription for pathogenic IL-17 production. In addition, IL-21 is a central cytokine for promotion of B cell antibody production. Fas (CD95)^−/−^ CREMα tg mice display enhanced anti-dsDNA autoantibodies and enhanced total IgG production (7, 8), which could possibly be related to this fact. Transcription of CREM itself is dependent on calcium/calmodulin-dependent protein kinase type IV (CamKIV) activation (46). Pharmacologic inhibition of CamKIV by the inhibitor KN93 or genetic inhibition of CamKIV decreases frequency of IL-17 producing cells and efficiently decreases lupus-like disease in murine models and ameliorates experimental autoimmune encephalomyelitis (47, 48).

We already demonstrated a pathogenic role for CREMα in lupus disease as well as in a model of acute lung injury (7, 49). Here, we analyzed CREMα tg T cells and IL-21 expression of these T cells within an acute model of murine colitis. Several observations indicate that IL-21 can contribute to the detrimental response in inflammatory bowel disease-related inflammation. IL-21 is overproduced in inflamed gut of patients with inflammatory bowel disease compared to non-inflamed and inflamed controls (11, 50). Furthermore, mice lacking IL-21 are unable to upregulate Th17-associated molecules during experimental gut inflammation and are largely protected against chemically induced colitis (29, 30). Moreover, WT mice, given a neutralizing IL-21R/Fc fusion protein, exhibit an amelioration of experimental colitis as compared to control mice (29); however, this was not confirmed by our experiments, which could be due to strain-specific alterations or due to the pharmacologic properties of the molecule. Nevertheless, how IL-21 is regulated in inflammatory bowel disease is not fully understood. A better understanding of the regulation of IL-21 expression in the context of inflammatory disease might be helpful in developing novel treatments to control disease pathogenesis.

Our data suggest that CREMα might be involved in IL-21 secretion of T cells in DSS-colitis as was shown before in a contact dermatitis model. CREMα expression in T cells from inflammatory bowel disease patients has not been investigated yet, further research will show if CREMα expression is elevated within T cells or inflamed tissues of these patients and if the CamKIV inhibitor KN-93 might also be of benefit in colitis.

## Author Contributions

KO and AW performed experiments and wrote the manuscript; RL cloned the CREM transgenic mice and performed experiments; AS performed experiments; CZ performed experiments; JR performed conceptual work; NW wrote the manuscript; and KT supervised and wrote the manuscript.

## Conflict of Interest Statement

The authors declare that the research was conducted in the absence of any commercial or financial relationships that could be construed as a potential conflict of interest.

## Abbreviations

CREM, cAMP-responsive element modulator; CSA, cyclosporine A; DSS, dextran sodium sulfate; *Il-21-luc*, IL-21 promoter luciferase plasmid; mLN, mesenterial lymph node; NP-CGG, 4-hydroxy-3-nitrophenylacetyl chicken gamma globulin; P/I, Phorbol-12-myristat-13-acetat/ionomycin.
