# Supplementary material for: CREM Alpha Enhances IL-21 Production in T Cells In Vivo and In Vitro
Source: Front Immunol. 2016 Dec 19;7:618. doi: 10.3389/fimmu.2016.00618 (PMC5165720; doi:10.3389/fimmu.2016.00618)
Supplement: Supplementary file 1 [file Image_1.pdf]

Supplemental Fig. 1

A

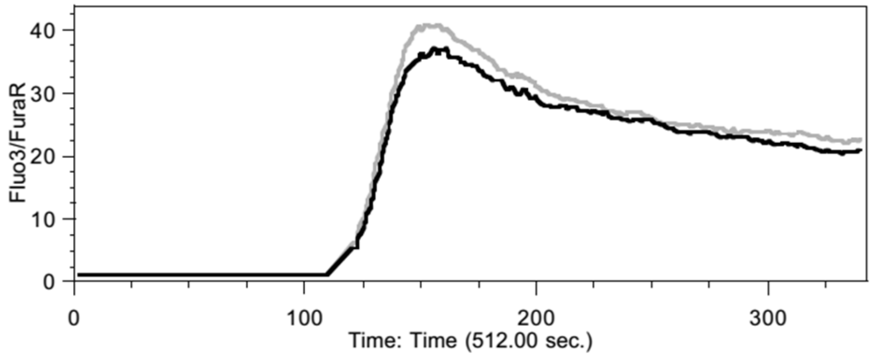

Pan-T cells were isolated via MACS-isolation from CREMα tg mice and appropriate wildtype mice. Ca^{2+} influx was measured immediately after stimulation with P/I. The picture shows a typical experiment (n=4)

B

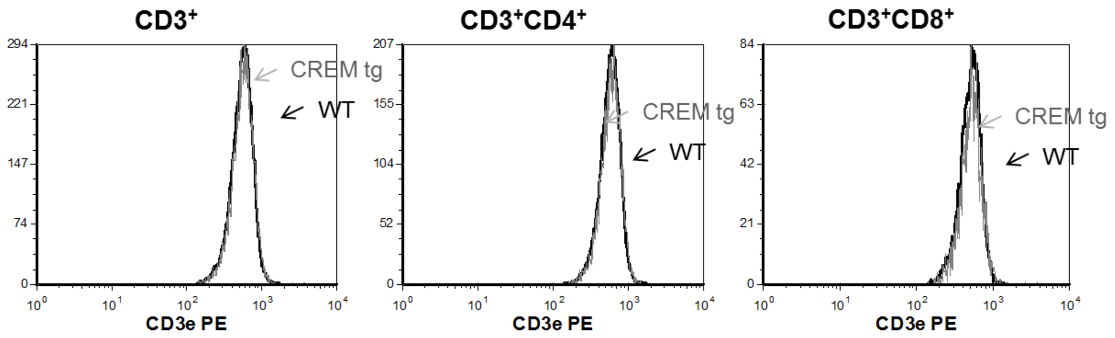

Representative histograms of CD3 ε expression in splenic T cells of WT (black) and CREMα tg (grey) mice, gated on $CD3^+$ (left), $CD3^+CD4^+$ (middle) and $CD3^+CD8^+$ (right) cells.
