# Supplementary material for: CREM Alpha Enhances IL-21 Production in T Cells In Vivo and In Vitro
Source: Front Immunol. 2016 Dec 19;7:618. doi: 10.3389/fimmu.2016.00618 (PMC5165720; doi:10.3389/fimmu.2016.00618)
Supplement: Supplementary file 2 [file Image_2.pdf]

Supplemental Fig. 2

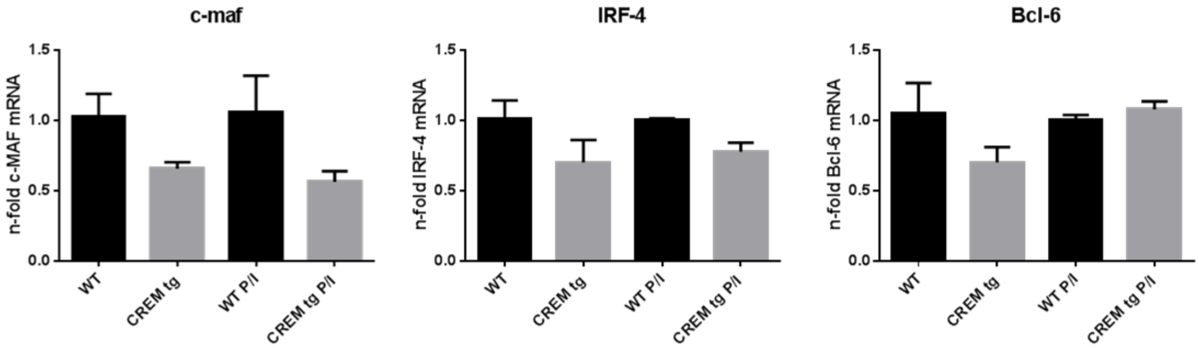

mRNA expression of CREMα tg T cells n-fold compared to WT cells. T cells were isolated and either left unstimulated or treated with P/I for 6 hours.
